# Distal and lateral subungual onychomycosis of the finger nail in a neonate: a rare case

**DOI:** 10.1186/s12887-019-1549-9

**Published:** 2019-05-27

**Authors:** Supram Hosuru Subramanya, Saujan Subedi, Yang Metok, Ajay Kumar, Peralam Yegneswaran Prakash, Niranjan Nayak

**Affiliations:** 1 Manipal College of Medical Sciences, Pokhara, Nepal; 2 Manipal Academy of Higher Education, Manipal, India

**Keywords:** *Candida* onychomycosis, Neonate, Nepal

## Abstract

**Background:**

Onychomycosis is extremely rare in neonates, infrequently reported in children and is considered to be exclusively a disease of adults.

**Case presentation:**

We, herein report a case of fingernail onychomycosis in a 28-day-old, healthy, male neonate. The child presented with a history of yellowish discoloration of the fingernail of the left hand for one week. The etiological agent was demonstrated both by microscopic examination and culture of nail clippings. The isolate grown on culture was identified as *Candida albicans* by phenotypic characteristics and by matrix-assisted laser desorption ionization-time of flight mass spectrometry. Antifungal sensitivity testing was performed by broth dilution method as per the Clinical & Laboratory Standards Institute guidelines. An oral swab culture of the child also yielded *C. albicans* with the same antibiogram as the nail isolate. The case was diagnosed as distal and lateral subungual *candida* onychomycosis of severity index score 22 (severe) and was treated with syrup fluconazole 6 mg/kg body weight/week and 5% amorolfine nail lacquer once/week for three months. After three months of therapy, the patient completely recovered with the development of a healthy nail plate.

**Conclusions:**

The case is presented due to its rarity in neonates which, we suppose is the first case report of onychomycosis from Nepal in a 28-day-old neonate. Oral colonization with pathogenic yeasts and finger suckling could be risk factors for neonatal onychomycosis.

## Background

Onychomycosis is a common nail plate infection caused by dermatophytes, non-dermatophytic molds, and yeasts. The prevalence of this condition is low in children as compared to adults and even rarer in the newborn [1]. Nevertheless, this diagnosis cannot be excluded in children, and neonates are presenting with nail plate disorders. *Candida* onychomycosis, most frequently caused by *Candida albicans,* rather than any other *Candida* species, often clinically presents as paronychia, onycholysis [2] and onychorrhexis. Onycholysis as a pathological entity in children is more commonly caused by dermatophytes and usually present with a single toenail infection having features of disto-lateral subungual onychomycosis [3]. Distal lateral subungual onychomycosis due to *Candida* is extremely uncommon in the neonates. We here describe a case of dorsal lateral onychomycosis of a fingernail due to *C. albicans* in a newborn baby. This is the first ever report of neonatal onychomycosis in Nepal, and the case is presented for its extreme rarity.

## Case presentation

The parents brought a 28-day-old male child to the Dermatology outpatient department of Manipal Teaching Hospital, Pokhara, Nepal with the complaints of yellowish discoloration of the nail with slight swelling of the upper part of the middle finger of the left hand for one week. Mother reported that the baby had been suckling this finger since birth. The baby was well two weeks back when he developed slight yellowish discoloration of the middle finger of the left hand. The stain spread proximally with increasing thickness of the nail. There was no family history of fungal infections, psoriasis, lichen planus, Darrier’s disease, or yellow nail syndrome. There were no other risk factors suggestive of HIV infection in the parents. The baby was delivered at 39 weeks of gestational age via normal vaginal delivery and weighed 3250 g. There was no history of perinatal hypoxia. His developmental milestones were appropriate for his age.

On examination, the physical activities of baby were as per his age. There was noticeable yellowish discoloration of the nail of the middle finger of the left hand distally with yellowish subungal hyperkeratotic debris. Pitting or whitish deposits on the nail were not evident. Examination of the skin revealed no lesions suggestive of fungal infections, psoriasis, lichen planus or Darrier’s disease. Scalp hairs were healthy. Oral and genital surfaces were normal without any lesions suggestive of mucosal candidiasis. Systemic examination was within normal limits. It was provisionally diagnosed as onychomycosis. The nail was trimmed, and parents were counselled to come for follow-up every month, keeping in view that the condition may be self-limiting. However, at two months follow up, increased discoloration and thickness of the nail without the involvement of glabrous skin was observed. Nail specimens (nail clippings) and oral swabs were sent for laboratory diagnosis.

### Investigations

Before collecting the sample, the nail and distal meta-phalangeal areas were thoroughly cleaned with alcohol to remove skin contaminants. The discolored portion of the nail was clipped; the nail bed and underside of the nail plate were scraped with the help of a sterile serrated curette after discarding the outermost debris. Avoiding injury to the nail plate and bleeding, as much of nail material as possible from the advancing infected edge closest to the cuticle, and the site close to the lateral nail edges was collected.

The material thus obtained was divided into four aliquots; one for direct microscopy using a 30% potassium hydroxide wet mount, second for fluorescent microscopy using calcofluor white, the third part was subjected to histopathological examination and, the fourth portion was cultured onto a set of two Sabouraud Dextrose Agar (SDA) with chloramphenicol. Swabs were also obtained from the oral cavity of the baby and were subjected to direct microscopy and culture.

**Fig. 1:**
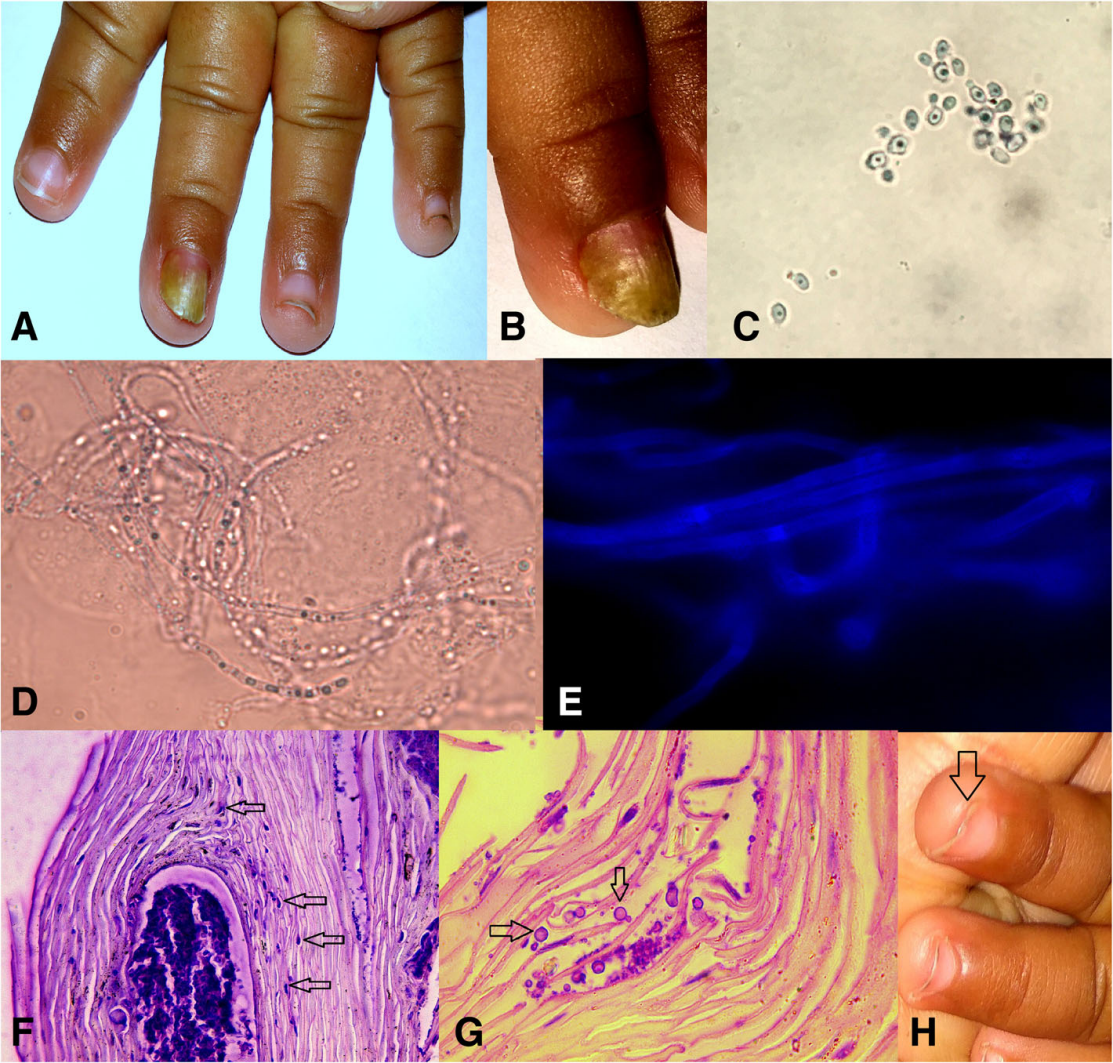
(**a**, **b**) White thickened infected nail on (DLSO) middle finger of the left hand involving half of the nail with subungual hyperkeratosis and onycholysis. (**c**, **d**) 30% KOH preparation showing pseudohyphae and yeast cells (1000X). (**e**) Fungal elements in calcofluor white staining under the fluorescent microscope (1000X). (**f**, **g**) H&E and PAS staining of the nail section showing yeast cells and pseudohyphae (arrow mark, 1000X), (**h**) Healthy nail plate after three months of therapy

The direct smear examinations and the histopathological examination revealed yeast cells with pseudohyphae (Fig. 1). *C. albicans* was isolated from the nail specimen and the oral swab. The isolates were identified by conventional methods and confirmed by MALDI-TOF mass spectrometry (matrix-assisted laser desorption ionization-time of flight mass spectrometry). Antifungal sensitivity testing of the isolates from the nail and oral cavity was performed by microbroth dilution method (Clinical & Laboratory Standards Institute guidelines). Both isolates had similar minimum inhibitory concentration (MIC) values (μg/mL): fluconazole (2), voriconazole (0.1), caspofungin(0.06), amphotericin B (1), and anidulafungin (0.03).

The lesion was graded to be of severity index 22 on the onychomycosis severity index scale [4]. It is considered as severe infection [area of involvement: 3, the proximity of disease to the matrix: 4, the presence of dermatophytoma (patch/streaks) or subungual hyperkeratosis (> 2 mm): 10]. Blood investigations such as complete blood count, liver function test, renal function test, random blood sugar and routine urinalysis were within normal limits. The child was treated with fluconazole syrup 6 mg/kg/week and 5% amorolfine nail lacquer once/week. After three months of therapy, the patient completely recovered with the development of a healthy nail plate.

## Discussion and conclusion

Onychomycosis is common in adults, with an increasing prevalence with age due to reduced nail growth accompanied by an increase in nail plate thickness [5–7]. Onychomycoses commonly affects toenails and is rarely seen in children. The rarity of this condition in pediatric patients has been attributed to the differences in nail plate structure, less exposure to trauma, and faster linear nail growth [5].

Etiological agents of onychomycosis include the dermatophytes *Trichophyton rubrum* and *Trichophyton mentagrophytes* [7, 8]. The role of *Candida* species in onychomycosis has also been established but is still primarily considered to cause onychomycosis secondary to paronychial disease or peripheral vascular disease [9–11]. According to earlier studies, out of all *Candida* species, *C. albicans* accounted for 57–87% of the infections [10, 12]. However, *C. albicans* causing nail infection in children and neonates are far less commonly encountered. The case is therefore reported for its rarity.

The present case was a 28-day-old healthy neonate with no predisposing condition in mother or neonate [13]. The mother was immunocompetent with no history of indwelling catheters. Neither the mother nor the neonate received broad-spectrum antibiotics or parenteral alimentation. Neonatal onychomycosis has been sparsely reported, and mainly ascribed to be due to *Candida tropicalis, Candida albicans, Candida parapsilosis* and *Trichophyton rubrum* [14]. The age range of reported cases with onychomycosis is 35 days to 10 weeks [14, 15]. Chun et al. [16] reported onychomycosis (an isolated lesion of the nail plate without the involvement of the glabrous skin caused by *C. tropicalis*) in a 107-day old infant. Our case had a clinical presentation similar to that documented by Chun et al. [16], except for the younger age. In spite of the discrepancies as mentioned above in the clinical presentation and pathology, *Candida* could be accounted as an important cause of onychomycosis in the pediatric age group. In a survey of 46 cases of onychomycosis in children, *Candida* was placed as the second causative agent of onychomycosis, next to dermatophytes [17]. Hay et al. [9] observed onychomycoses in subjects following chronic mucocutaneous candidiasis and chronic paronychia. Carvalho VO, et al. [1] documented childhood onychomycosis due to *Fusarium oxysporum*, which was acquired in-utero. It was a progressing, ascending infection in an HIV positive mother involving the placenta and nail as proved by molecular methods. Congenital candidiasis, acquired transplacentally, as shown earlier [1], might present with skin rash, but also could affect skin appendages as reported in the past [18]. The present neonatal distal lateral subungual onychomycosis without any predisposing conditions either in the baby or the mother is a rarity.

*Candida* onychomycosis in infants and neonates without any predisposition is rare. Sanchez-Schmidt et al. [19] have reported nail changes suggestive of onychomycoses appearing between 2 and 6 weeks of life without any signs of immunosuppression in any of their cases. Histopathological shreds of evidence, such as distal lateral subungual lesions with onycholysis, as seen in our case are similar to those showed by Hay et al. [9]. He noticed three patterns of nail diseases due to *Candida*: total dystrophic onychomycosis, mostly seen in chronic mucocutaneous candidiasis; proximal and lateral nail dystrophy, secondary to chronic paronychia; and distal and lateral nail dystrophy, associated with onycholysis, sloughing of the nail with peripheral vascular disease, and finger and toenail abnormalities.

Finger suckling is a prevalent behavior in neonates and infants. It can lead to maceration of the digits and increase the risk of transmission of oral flora to the hyponychium and nail folds. Isolation of phenotypically matching (similar MIC values) *C. albicans* from oral swab and nail bed in the present case suggests a high possibility that the organisms from the oral cavity might have colonized the nail leading to infection. The maternal screening for vaginal candidiasis would be important to trace the source of infection, as it was not done in the present case. Further justification would only be possible by molecular typing of the isolates.

In this case, the clinical diagnosis of onychomycosis was made during the first visit itself. Thus the patient was advised to come for follow up with nail trimming, without any antifungal use, with the assumption that there would be a spontaneous resolution in a few months, because of the faster linear growth of the nail in children.

We have reported a case of onychomycosis caused by *C. albicans* in a neonate without any evidence of a compromised immune system in the mother or the baby. Accurate identification of the causative agent and timely initiation of antifungal therapy led to complete recovery. To the best of our knowledge, this is the first case report of onychomycosis due to *C. albicans* at such a young age. Oral colonization with pathogenic yeasts and finger suckling could be risk factors for neonatal onychomycosis.

## Data Availability

Not applicable.

## Abbreviations

CLSI: The Clinical Laboratory Standards Institute
HIV: Human Immunodeficiency Virus
MALDI-TOF mass spectrometry: Matrix-assisted laser desorption ionization-time of flight mass spectrometry
MIC: Minimum Inhibitory Concentration
SDA: Sabouraud Dextrose Agar
